# Crystal structure of 2-(adamantan-1-yl)-5-(4-bromo­phen­yl)-1,3,4-oxa­diazole

**DOI:** 10.1107/S1600536814023861

**Published:** 2014-11-05

**Authors:** Nourah Z. Alzoman, Ali A. El-Emam, Hazem A. Ghabbour, C. S. Chidan Kumar, Hoong-Kun Fun

**Affiliations:** a Department of Pharmaceutical Chemistry, College of Pharmacy, King Saud University, PO Box 2457, Riaydh 11451, Saudi Arabia; b King Abdullah Institute for Nanotechnology (KAIN), King Saud University, Riyadh 11451, Saudi Arabia; c X-ray Crystallography Unit, School of Physics, Universiti Sains Malaysia, 11800 USM, Penang, Malaysia; d Department of Chemistry, Alva’s Institute of Engineering & Technology, Mijar, Moodbidri 574225, Karnataka, India

**Keywords:** crystal structure, adamntane derivative, 1,3,4-oxa­diazole, C—H⋯π hydrogen bonds, π–π inter­actions

## Abstract

In the title mol­ecule, C_18_H_19_BrN_2_O, the benzene ring is inclined to the oxa­diazole ring by 10.44 (8)°. In the crystal, C—H⋯π inter­actions link the mol­ecules in a head-to-tail fashion, forming chains extending along the *c-*axis direction. The chains are further connected by π–π stacking inter­actions, with centroid–centroid distances of 3.6385 (7) Å, forming layers parallel to the *bc* plane.

## Related literature   

For the biological activity of adamantane derivatives, see: Al-Abdullah *et al.* (2014 ▶); Vernier *et al.* (1969 ▶); El-Emam *et al.* (2013 ▶); Kadi *et al.* (2010 ▶); Balzarini *et al.* (2009 ▶). For the biological activity of adamantyl-1,3,4- oxa­diazole derivatives, see: Al-Deeb *et al.* (2006 ▶); El-Emam *et al.* (2004 ▶); Kadi *et al.* (2007 ▶). For related adamantyl 1,3,4-oxa­diazole structures, see: El-Emam *et al.* (2012 ▶); Al-Omary *et al.* (2014 ▶). For related 2,5-disubstituted 1,3,4-oxa­diazole structures, see: Cordes *et al.* (2011 ▶); Franco *et al.* (2003 ▶). For the synthesis of the title compound, see: Kadi *et al.* (2007 ▶).
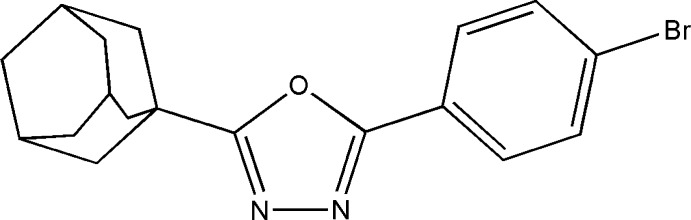


## Experimental   

### Crystal data  

C_18_H_19_BrN_2_O
*M* *_r_* = 359.26
Monoclinic, 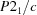

*a* = 13.2571 (5) Å
*b* = 6.4753 (3) Å
*c* = 19.6761 (7) Å
β = 114.924 (2)°
*V* = 1531.76 (11) Å^3^
*Z* = 4
Mo *K*α radiation
μ = 2.69 mm^−1^
*T* = 293 K
0.28 × 0.22 × 0.10 mm

### Data collection  

Bruker APEXII CCD diffractometer
Absorption correction: multi-scan (*SADABS*; Bruker, 2009 ▶) *T*_min_ = 0.520, *T*_max_ = 0.779
39946 measured reflections
4678 independent reflections
3996 reflections with *I* > 2σ(*I*)
*R*_int_ = 0.033

### Refinement  

*R*[*F* ^2^ > 2σ(*F* ^2^)] = 0.031
*wR*(*F* ^2^) = 0.069
*S* = 1.06
4678 reflections
199 parameters
H-atom parameters constrained
Δρ_max_ = 0.40 e Å^−3^
Δρ_min_ = −0.50 e Å^−3^

### 

Data collection: *APEX2* (Bruker, 2009 ▶); cell refinement: *SAINT* (Bruker, 2009 ▶); data reduction: *SAINT*; program(s) used to solve structure: *SHELXS97* (Sheldrick, 2008 ▶); program(s) used to refine structure: *SHELXL97* (Sheldrick, 2008 ▶); molecular graphics: *SHELXTL* (Sheldrick, 2008 ▶); software used to prepare material for publication: *SHELXTL* and *PLATON* (Spek, 2009 ▶).

## Supplementary Material

Crystal structure: contains datablock(s) global, I. DOI: 10.1107/S1600536814023861/rz5137sup1.cif

Structure factors: contains datablock(s) I. DOI: 10.1107/S1600536814023861/rz5137Isup2.hkl

Supporting information file. DOI: 10.1107/S1600536814023861/rz5137Isup3.cml

**Fig. 1.**
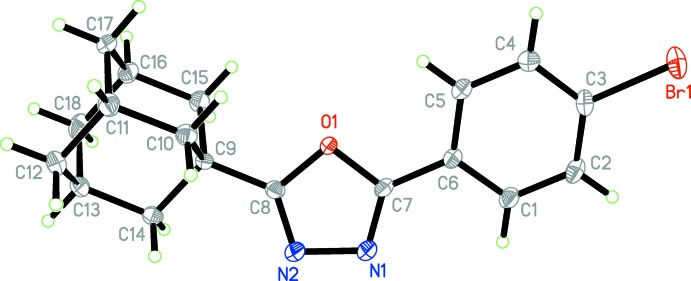
The molecular structure of the title compound with 50% probability displacement ellipsoids.

. DOI: 10.1107/S1600536814023861/rz5137fig1.tif
The mol­ecular structure of the title compound with 50% probability displacement ellipsoids.

**Fig. 2.**
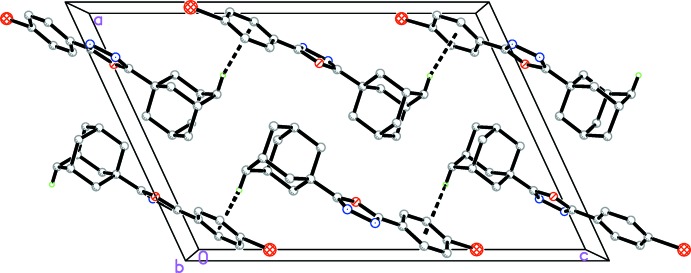
Crystal packing of the title compound, showing the C–H···π interactions as dashed lines. Other H-atoms are omitted for clarity.

. DOI: 10.1107/S1600536814023861/rz5137fig2.tif
Crystal packing of the title compound, showing the C–H⋯π inter­actions as dashed lines. Other H-atoms are omitted for clarity.

CCDC reference: 1031604

Additional supporting information:  crystallographic information; 3D view; checkCIF report

## Figures and Tables

**Table 1:** Hydrogen-bond geometry (, ) *Cg*1 is the centroid of the C1C6 ring.

*D*H*A*	*D*H	H*A*	*D* *A*	*D*H*A*
C18H18*B* *Cg*1^i^	0.97	2.74	3.6709(19)	162

Symmetry code: (i) 
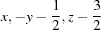
.

## supplementary crystallographic information

### S1. Comment

Adamantane derivatives have long been known for their diverse biological activities including antiviral activity against the influenza (Vernier *et al.*, 1969) and HIV viruses (El-Emam *et al.*, 2004; Balzarini *et al.*, 2009). In addition, Adamantyl 1,3,4-oxadiazole derivative were reported to exhibit marked antibacterial and anti-inflammatory activities (Kadi *et al.*, 2007, 2010). In continuation to our interest in the chemical and structural properties of adamantane derivatives (El-Emam *et al.*, 2012; Al-Omary *et al.*, 2014) the title compound (I) was prepared as potential bioactive agent.

In the title compound (Fig. 1), the benzene (C1–C6) ring is inclined relative to the oxadiazole (O1/N1/N2/C7/C8) ring by a dihedral angle of 10.44 (8)%. Bond lengths (Allen *et al.*, 1987) and angles in the title compound are within normal ranges and are comparable with those reported earlier for the structure of related compounds (Cordes *et al.*, 2011; Franco *et al.*, 2003). In the crystal structure, the molecules are connected into head-to-tail fashion to form chains extending along the *c* axis *via* C–H···π interactions (Table 1, Fig. 2) involving the centroid of the C1–C6 benzene ring (*Cg*1). In addition, π–π interactions (*Cg*1··· *Cg*1^i^ = 3.6385 (7) Å; symmetry code: (i) -x, -y, 1-z) link the chains into layers parallel to the *bc* plane.

### S2. Experimental

The title compound was prepared following our previously described method (Kadi *et al.*, 2007). A mixture of the 4-bromobenzoic acid hydrazide (2.15 g, 0.01 mol), 1-adamantane carboxylic acid (1.8 g, 0.01 mol) and phosphorus oxychloride (8 ml) was heated under reflux for 1 h. On cooling, crushed ice (50 g) was added cautiously and the mixture was stirred for 30 min. The separated crude product was filtered, washed with water, then with a saturated sodium hydrogen carbonate solution and finally with water, dried and crystallized from EtOH/CHCl_3_ (1:1 *v*/*v*) to yield 3.16 g (88%) of the title compound (C_18_H_19_BrN_2_O) as colorless crystals. M. p.: 188–190 °C.

^1^H NMR (CDCl_3_): δ 1.81 (s, 6H, Adamantane-H), 2.15 (s, 9H, Adamantane-H), 7.64 (d, 2H, Ar—H, J = 8.1 Hz), 7.92 (d, 2H, Ar—H, J = 8.1 Hz). 13 C NMR: δ 27.74, 34.45, 36.29, 39.96 (Adamantane-C), 123.26, 125.99, 128.23, 132.28 (Ar—C), 163.56 (Oxadiazole C-5), 172.85 (Oxadiazole C-2).

### S3. Refinement

All the H atoms were positioned geometrically (C=H 0.93–0.98 Å) and refined using a riding model with *U*_iso_(H) = 1.2 *U*_eq_(C).

### Crystal data

**Table:**

C_18_H_19_BrN_2_O	*F*(000) = 736
*M**_r_* = 359.26	*D*_x_ = 1.558 Mg m^−^^3^
Monoclinic, *P*2_1_/*c*	Mo *K*α radiation, λ = 0.71073 Å
Hall symbol: -P 2ybc	Cell parameters from 9861 reflections
*a* = 13.2571 (5) Å	θ = 2.3–30.5°
*b* = 6.4753 (3) Å	µ = 2.69 mm^−^^1^
*c* = 19.6761 (7) Å	*T* = 293 K
β = 114.924 (2)°	Block, colourless
*V* = 1531.76 (11) Å^3^	0.28 × 0.22 × 0.10 mm
*Z* = 4	

### Data collection

**Table:**

Bruker APEXII CCD diffractometer	4678 independent reflections
Radiation source: fine-focus sealed tube	3996 reflections with *I* > 2σ(*I*)
Graphite monochromator	*R*_int_ = 0.033
φ and ω scans	θ_max_ = 30.6°, θ_min_ = 2.3°
Absorption correction: multi-scan (*SADABS*; Bruker, 2009)	*h* = −18→18
*T*_min_ = 0.520, *T*_max_ = 0.779	*k* = −9→9
39946 measured reflections	*l* = −28→28

### Refinement

**Table:**

Refinement on *F*^2^	Primary atom site location: structure-invariant direct methods
Least-squares matrix: full	Secondary atom site location: difference Fourier map
*R*[*F*^2^ > 2σ(*F*^2^)] = 0.031	Hydrogen site location: inferred from neighbouring sites
*wR*(*F*^2^) = 0.069	H-atom parameters constrained
*S* = 1.06	*w* = 1/[σ^2^(*F*_o_^2^) + (0.0261*P*)^2^ + 1.1502*P*] where *P* = (*F*_o_^2^ + 2*F*_c_^2^)/3
4678 reflections	(Δ/σ)_max_ = 0.001
199 parameters	Δρ_max_ = 0.40 e Å^−^^3^
0 restraints	Δρ_min_ = −0.50 e Å^−^^3^

### Special details

**Table:**

Geometry. All e.s.d.'s (except the e.s.d. in the dihedral angle between two l.s. planes) are estimated using the full covariance matrix. The cell e.s.d.'s are taken into account individually in the estimation of e.s.d.'s in distances, angles and torsion angles; correlations between e.s.d.'s in cell parameters are only used when they are defined by crystal symmetry. An approximate (isotropic) treatment of cell e.s.d.'s is used for estimating e.s.d.'s involving l.s. planes.
Refinement. Refinement of *F*^2^ against ALL reflections. The weighted *R*-factor *wR* and goodness of fit *S* are based on *F*^2^, conventional *R*-factors *R* are based on *F*, with *F* set to zero for negative *F*^2^. The threshold expression of *F*^2^ > σ(*F*^2^) is used only for calculating *R*-factors(gt) *etc*. and is not relevant to the choice of reflections for refinement. *R*-factors based on *F*^2^ are statistically about twice as large as those based on *F*, and *R*- factors based on ALL data will be even larger.

### Fractional atomic coordinates and isotropic or equivalent isotropic displacement parameters (Å^2^)

**Table:**

	*x*	*y*	*z*	*U*_iso_*/*U*_eq_	
Br1	0.032308 (13)	−0.26186 (3)	0.704343 (9)	0.02674 (6)	
O1	0.23225 (9)	0.17552 (15)	0.46715 (6)	0.0156 (2)	
N1	0.16178 (12)	0.4626 (2)	0.48948 (8)	0.0232 (3)	
N2	0.21377 (12)	0.5092 (2)	0.44146 (8)	0.0220 (3)	
C1	0.06684 (12)	0.2219 (2)	0.57759 (8)	0.0182 (3)	
H1A	0.0403	0.3560	0.5652	0.022*	
C2	0.03445 (12)	0.1033 (3)	0.62379 (8)	0.0204 (3)	
H2A	−0.0139	0.1568	0.6425	0.025*	
C3	0.07532 (12)	−0.0960 (2)	0.64159 (8)	0.0184 (3)	
C4	0.14611 (13)	−0.1809 (2)	0.61362 (8)	0.0190 (3)	
H4A	0.1722	−0.3153	0.6260	0.023*	
C5	0.17744 (12)	−0.0626 (2)	0.56699 (8)	0.0184 (3)	
H5A	0.2240	−0.1184	0.5472	0.022*	
C6	0.13929 (12)	0.1400 (2)	0.54964 (8)	0.0150 (3)	
C7	0.17477 (12)	0.2668 (2)	0.50242 (8)	0.0155 (3)	
C8	0.25323 (12)	0.3371 (2)	0.43018 (8)	0.0149 (3)	
C9	0.32131 (11)	0.2945 (2)	0.38782 (7)	0.0129 (2)	
C10	0.43936 (12)	0.2361 (2)	0.44483 (8)	0.0163 (3)	
H10A	0.4702	0.3473	0.4807	0.020*	
H10B	0.4361	0.1132	0.4720	0.020*	
C11	0.51455 (12)	0.1961 (2)	0.40472 (9)	0.0192 (3)	
H11A	0.5896	0.1613	0.4417	0.023*	
C12	0.51921 (13)	0.3889 (2)	0.36103 (9)	0.0220 (3)	
H12A	0.5668	0.3634	0.3356	0.026*	
H12B	0.5501	0.5036	0.3953	0.026*	
C13	0.40153 (13)	0.4430 (2)	0.30350 (9)	0.0202 (3)	
H13A	0.4046	0.5659	0.2753	0.024*	
C14	0.32711 (13)	0.4874 (2)	0.34404 (8)	0.0182 (3)	
H14A	0.2530	0.5245	0.3078	0.022*	
H14B	0.3572	0.6025	0.3783	0.022*	
C15	0.27356 (12)	0.1114 (2)	0.33328 (8)	0.0181 (3)	
H15A	0.2698	−0.0105	0.3608	0.022*	
H15B	0.1989	0.1439	0.2968	0.022*	
C16	0.34879 (13)	0.0691 (2)	0.29311 (9)	0.0210 (3)	
H16A	0.3185	−0.0468	0.2583	0.025*	
C17	0.46678 (13)	0.0156 (2)	0.35028 (9)	0.0217 (3)	
H17A	0.4650	−0.1076	0.3778	0.026*	
H17B	0.5137	−0.0117	0.3245	0.026*	
C18	0.35327 (14)	0.2619 (3)	0.24913 (9)	0.0240 (3)	
H18A	0.3994	0.2350	0.2228	0.029*	
H18B	0.2790	0.2962	0.2124	0.029*	

### Atomic displacement parameters (Å^2^)

**Table:**

	*U*^11^	*U*^22^	*U*^33^	*U*^12^	*U*^13^	*U*^23^
Br1	0.02327 (9)	0.03737 (10)	0.02322 (8)	−0.00111 (7)	0.01336 (6)	0.00809 (7)
O1	0.0214 (5)	0.0128 (4)	0.0179 (5)	0.0028 (4)	0.0133 (4)	0.0014 (4)
N1	0.0332 (7)	0.0170 (6)	0.0291 (7)	0.0049 (5)	0.0227 (6)	0.0016 (5)
N2	0.0305 (7)	0.0158 (6)	0.0276 (7)	0.0048 (5)	0.0201 (6)	0.0023 (5)
C1	0.0185 (6)	0.0197 (7)	0.0187 (6)	0.0043 (5)	0.0101 (5)	0.0008 (5)
C2	0.0185 (7)	0.0274 (8)	0.0183 (7)	0.0044 (6)	0.0107 (6)	−0.0005 (6)
C3	0.0167 (6)	0.0261 (7)	0.0136 (6)	−0.0029 (6)	0.0076 (5)	0.0011 (5)
C4	0.0206 (7)	0.0174 (6)	0.0202 (7)	0.0021 (6)	0.0096 (6)	0.0013 (5)
C5	0.0196 (7)	0.0186 (7)	0.0205 (7)	0.0028 (5)	0.0118 (6)	−0.0005 (5)
C6	0.0157 (6)	0.0166 (6)	0.0135 (6)	0.0013 (5)	0.0068 (5)	−0.0013 (5)
C7	0.0164 (6)	0.0160 (6)	0.0163 (6)	0.0025 (5)	0.0089 (5)	−0.0018 (5)
C8	0.0168 (6)	0.0129 (6)	0.0150 (6)	0.0003 (5)	0.0067 (5)	0.0010 (5)
C9	0.0147 (6)	0.0108 (6)	0.0139 (6)	0.0006 (5)	0.0068 (5)	0.0005 (4)
C10	0.0164 (6)	0.0165 (6)	0.0152 (6)	0.0014 (5)	0.0060 (5)	0.0021 (5)
C11	0.0147 (6)	0.0208 (7)	0.0223 (7)	0.0026 (5)	0.0081 (6)	0.0046 (6)
C12	0.0221 (7)	0.0193 (7)	0.0296 (8)	−0.0039 (6)	0.0158 (6)	0.0005 (6)
C13	0.0269 (8)	0.0165 (6)	0.0226 (7)	0.0027 (6)	0.0156 (6)	0.0064 (5)
C14	0.0224 (7)	0.0137 (6)	0.0212 (7)	0.0042 (5)	0.0117 (6)	0.0050 (5)
C15	0.0176 (7)	0.0185 (7)	0.0193 (7)	−0.0047 (5)	0.0089 (6)	−0.0057 (5)
C16	0.0270 (8)	0.0188 (7)	0.0224 (7)	−0.0040 (6)	0.0153 (6)	−0.0074 (6)
C17	0.0279 (8)	0.0150 (6)	0.0314 (8)	0.0052 (6)	0.0214 (7)	0.0029 (6)
C18	0.0278 (8)	0.0306 (8)	0.0173 (7)	0.0027 (7)	0.0130 (6)	0.0013 (6)

### Geometric parameters (Å, º)

**Table:**

Br1—C3	1.8965 (14)	C10—H10B	0.9700
O1—C7	1.3629 (16)	C11—C17	1.530 (2)
O1—C8	1.3682 (17)	C11—C12	1.532 (2)
N1—C7	1.2902 (19)	C11—H11A	0.9800
N1—N2	1.4169 (18)	C12—C13	1.533 (2)
N2—C8	1.2891 (18)	C12—H12A	0.9700
C1—C2	1.389 (2)	C12—H12B	0.9700
C1—C6	1.3968 (19)	C13—C18	1.534 (2)
C1—H1A	0.9300	C13—C14	1.535 (2)
C2—C3	1.386 (2)	C13—H13A	0.9800
C2—H2A	0.9300	C14—H14A	0.9700
C3—C4	1.386 (2)	C14—H14B	0.9700
C4—C5	1.386 (2)	C15—C16	1.536 (2)
C4—H4A	0.9300	C15—H15A	0.9700
C5—C6	1.396 (2)	C15—H15B	0.9700
C5—H5A	0.9300	C16—C17	1.533 (2)
C6—C7	1.4584 (19)	C16—C18	1.534 (2)
C8—C9	1.4899 (19)	C16—H16A	0.9800
C9—C14	1.5376 (19)	C17—H17A	0.9700
C9—C10	1.5397 (19)	C17—H17B	0.9700
C9—C15	1.5447 (19)	C18—H18A	0.9700
C10—C11	1.532 (2)	C18—H18B	0.9700
C10—H10A	0.9700		
			
C7—O1—C8	102.80 (11)	C12—C11—H11A	109.6
C7—N1—N2	106.19 (12)	C11—C12—C13	109.36 (12)
C8—N2—N1	106.11 (12)	C11—C12—H12A	109.8
C2—C1—C6	120.09 (14)	C13—C12—H12A	109.8
C2—C1—H1A	120.0	C11—C12—H12B	109.8
C6—C1—H1A	120.0	C13—C12—H12B	109.8
C3—C2—C1	118.96 (13)	H12A—C12—H12B	108.3
C3—C2—H2A	120.5	C12—C13—C18	109.63 (13)
C1—C2—H2A	120.5	C12—C13—C14	109.63 (12)
C4—C3—C2	121.79 (14)	C18—C13—C14	109.58 (13)
C4—C3—Br1	118.26 (12)	C12—C13—H13A	109.3
C2—C3—Br1	119.93 (11)	C18—C13—H13A	109.3
C3—C4—C5	119.04 (14)	C14—C13—H13A	109.3
C3—C4—H4A	120.5	C13—C14—C9	109.50 (11)
C5—C4—H4A	120.5	C13—C14—H14A	109.8
C4—C5—C6	120.16 (13)	C9—C14—H14A	109.8
C4—C5—H5A	119.9	C13—C14—H14B	109.8
C6—C5—H5A	119.9	C9—C14—H14B	109.8
C5—C6—C1	119.93 (13)	H14A—C14—H14B	108.2
C5—C6—C7	120.28 (13)	C16—C15—C9	109.24 (12)
C1—C6—C7	119.80 (13)	C16—C15—H15A	109.8
N1—C7—O1	112.48 (13)	C9—C15—H15A	109.8
N1—C7—C6	128.83 (13)	C16—C15—H15B	109.8
O1—C7—C6	118.67 (12)	C9—C15—H15B	109.8
N2—C8—O1	112.42 (12)	H15A—C15—H15B	108.3
N2—C8—C9	129.92 (13)	C17—C16—C18	109.12 (13)
O1—C8—C9	117.56 (12)	C17—C16—C15	110.23 (12)
C8—C9—C14	110.37 (11)	C18—C16—C15	109.44 (13)
C8—C9—C10	107.90 (11)	C17—C16—H16A	109.3
C14—C9—C10	109.40 (11)	C18—C16—H16A	109.3
C8—C9—C15	111.30 (11)	C15—C16—H16A	109.3
C14—C9—C15	109.70 (12)	C11—C17—C16	109.56 (12)
C10—C9—C15	108.11 (11)	C11—C17—H17A	109.8
C11—C10—C9	110.40 (11)	C16—C17—H17A	109.8
C11—C10—H10A	109.6	C11—C17—H17B	109.8
C9—C10—H10A	109.6	C16—C17—H17B	109.8
C11—C10—H10B	109.6	H17A—C17—H17B	108.2
C9—C10—H10B	109.6	C13—C18—C16	109.45 (12)
H10A—C10—H10B	108.1	C13—C18—H18A	109.8
C17—C11—C10	108.83 (12)	C16—C18—H18A	109.8
C17—C11—C12	109.40 (13)	C13—C18—H18B	109.8
C10—C11—C12	109.87 (12)	C16—C18—H18B	109.8
C17—C11—H11A	109.6	H18A—C18—H18B	108.2
C10—C11—H11A	109.6		
			
C7—N1—N2—C8	−0.10 (18)	O1—C8—C9—C15	52.62 (16)
C6—C1—C2—C3	0.1 (2)	C8—C9—C10—C11	−178.46 (11)
C1—C2—C3—C4	−1.0 (2)	C14—C9—C10—C11	−58.36 (15)
C1—C2—C3—Br1	−179.84 (11)	C15—C9—C10—C11	61.06 (15)
C2—C3—C4—C5	0.4 (2)	C9—C10—C11—C17	−61.10 (15)
Br1—C3—C4—C5	179.30 (11)	C9—C10—C11—C12	58.67 (16)
C3—C4—C5—C6	1.0 (2)	C17—C11—C12—C13	60.01 (16)
C4—C5—C6—C1	−1.8 (2)	C10—C11—C12—C13	−59.40 (16)
C4—C5—C6—C7	178.16 (14)	C11—C12—C13—C18	−59.80 (16)
C2—C1—C6—C5	1.3 (2)	C11—C12—C13—C14	60.53 (16)
C2—C1—C6—C7	−178.74 (14)	C12—C13—C14—C9	−60.59 (16)
N2—N1—C7—O1	0.28 (18)	C18—C13—C14—C9	59.77 (16)
N2—N1—C7—C6	178.56 (14)	C8—C9—C14—C13	177.65 (12)
C8—O1—C7—N1	−0.34 (16)	C10—C9—C14—C13	59.08 (15)
C8—O1—C7—C6	−178.81 (12)	C15—C9—C14—C13	−59.35 (15)
C5—C6—C7—N1	−168.91 (16)	C8—C9—C15—C16	−178.05 (12)
C1—C6—C7—N1	11.1 (2)	C14—C9—C15—C16	59.51 (15)
C5—C6—C7—O1	9.3 (2)	C10—C9—C15—C16	−59.72 (15)
C1—C6—C7—O1	−170.73 (13)	C9—C15—C16—C17	59.96 (16)
N1—N2—C8—O1	−0.12 (17)	C9—C15—C16—C18	−60.05 (16)
N1—N2—C8—C9	−176.25 (14)	C10—C11—C17—C16	59.51 (15)
C7—O1—C8—N2	0.27 (16)	C12—C11—C17—C16	−60.55 (15)
C7—O1—C8—C9	176.93 (12)	C18—C16—C17—C11	60.45 (15)
N2—C8—C9—C14	−9.4 (2)	C15—C16—C17—C11	−59.76 (16)
O1—C8—C9—C14	174.68 (12)	C12—C13—C18—C16	59.93 (16)
N2—C8—C9—C10	110.13 (17)	C14—C13—C18—C16	−60.43 (16)
O1—C8—C9—C10	−65.84 (15)	C17—C16—C18—C13	−60.03 (16)
N2—C8—C9—C15	−131.41 (16)	C15—C16—C18—C13	60.66 (16)

### Hydrogen-bond geometry (Å, º)

*Cg*1 is the centroid of the C1–C6 ring.

**Table:**

*D*—H···*A*	*D*—H	H···*A*	*D*···*A*	*D*—H···*A*
C18—H18*B*···*Cg*1^i^	0.97	2.74	3.6709 (19)	162

Symmetry code: (i) *x*, −*y*−1/2, *z*−3/2.
