# Supplementary figures and images for: Crystal structure of 2-(adamantan-1-yl)-5-(4-bromo­phen­yl)-1,3,4-oxa­diazole
Source: Acta Crystallogr Sect E Struct Rep Online. 2014 Nov 5;70(Pt 12):o1231–2. doi: 10.1107/S1600536814023861 (PMC4257436; doi:10.1107/S1600536814023861)

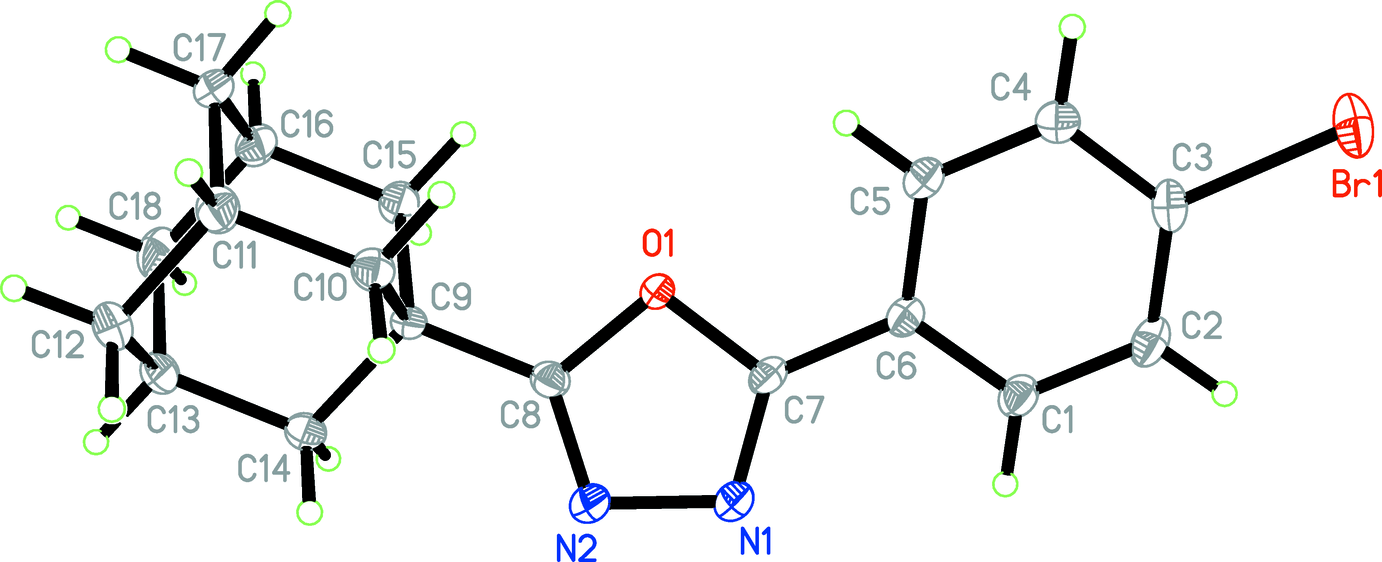

Supplement: Supplementary file 4 [file e-70-o1231-fig1.tif]

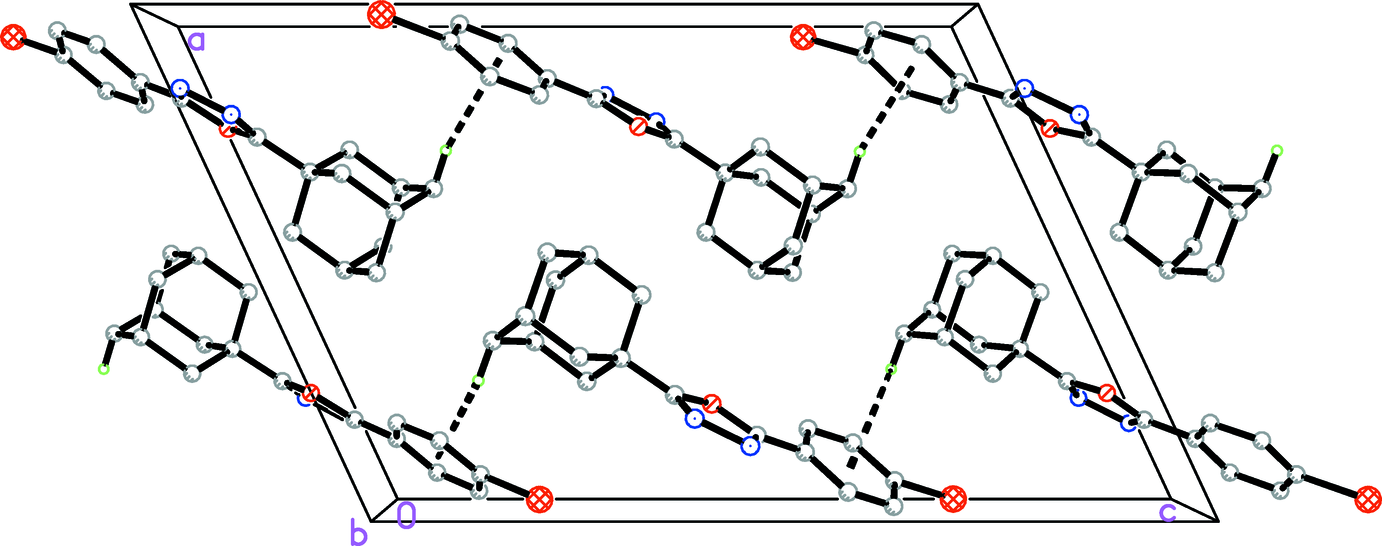

Supplement: Supplementary file 5 [file e-70-o1231-fig2.tif]
